# Influence of Diet on Bioaccessibility of Iron from Dietary Supplements and Medicinal Products—Results of In Vitro Digestion Model and Analytical Determinations

**DOI:** 10.3390/nu18081219

**Published:** 2026-04-13

**Authors:** Piotr Bawiec, Agnieszka Jaworowska, Jan Sawicki, Marcin Czop, Joanna Tokarczyk, Paweł Helon, Wojciech Koch

**Affiliations:** 1 Department of Food and Nutrition, Medical University of Lublin, 4a Chodźki Str., 20-093 Lublin, Poland; piotr.bawiec@wp.pl (P.B.); agnieszka.jaworowska@umlub.edu.pl (A.J.); joanna.tokarczyk@umlub.edu.pl (J.T.); 2 Department of Analytical Chemistry, Medical University of Lublin, 4a Chodźki Str., 20-093 Lublin, Poland; jan.sawicki@umlub.edu.pl; 3 Department of Clinical Genetics, Medical University of Lublin, Radziwiłłowska 11 Str., 20-080 Lublin, Poland; marcin.czop@umlub.edu.pl; 4 Branch in Sandomierz, Jan Kochanowski University of Kielce, Schinzla 13a Str., 27-600 Sandomierz, Poland; pawel.helon@ujk.edu.pl

**Keywords:** iron, bioaccessibility, dietary supplements, in vitro digestion

## Abstract

Background/Objectives: Iron is a key micronutrient for the proper growth and development of the organism. The aim of this study was to evaluate the impact of diet type, the chemical form of iron, and the formulation of the pharmaceutical preparation on its relative bioaccessibility from selected dietary supplements and medicinal products. Methods: The research was conducted using a two-stage in vitro digestion model, simulating the physiological processes occurring in the human digestive system and ICP-OES determination of iron. The analytical model used in the study involved homogenates of whole-day dietary rations (basic, standard, and high-residue diets) with the addition of selected dietary supplements or medicinal products. It was demonstrated that iron bioaccessibility was strictly determined by dietary composition and the chemical form of the preparation. Results: In the studies conducted without external supplementation, the highest iron bioaccessibility was observed in the basic diet model (7.96%), and the lowest in the standard diet (4.63%). The highest bioaccessibility value was determined for iron sulfate registered as medicine (12.58%), whereas the lowest was iron lactate (5.25%). The extended-release tablets observed the highest bioaccessibility (19.31%). Conclusions: It was proven that the developed in vitro digestion model may serve as an effective tool for the preliminary assessment of iron bioaccessibility, enabling the optimization of supplementation without ethical barriers.

## 1. Introduction

Iron (Fe) is one of the most important micronutrients, as it is essential for the proper growth and development of the body. The physiological supply of iron to the organism is limited to two sources: the placenta during the fetal period and the epithelium of the small intestine in later stages of life after birth [1]. The physiological concentration of the element in the serum is approximately 3–5 g of iron, of which about 60% is found in hemoglobin, 10% in myoglobin, and 1% in tissue enzymes such as cytochromes, catalases, or peroxidases. The remaining amount consists of protein complexes, primarily in the form of ferritin and hemosiderin. These constitute the so-called storage pool, located in the liver, spleen, and bone marrow [2,3].

The proper course of key biochemical reactions is strictly dependent on the availability of iron in the human body. Under conditions of homeostasis, iron transport is tightly controlled and occurs via transferrin. A single plasma protein molecule can transport a maximum of two iron atoms, and in this form, the molecules exhibit the highest affinity for receptors on the cell surface [4,5]. Although iron metabolism, unlike that of other metals, lacks a physiological excretion mechanism, iron losses still occur. These include mandatory losses through the skin, intestines, urinary tract, and respiratory tract, as well as menstrual blood loss in women of reproductive age [6].

Maintaining stable iron levels is crucial for the proper functioning of the body. A worsening iron deficiency leads to the development of anemia, manifesting as chronic fatigue and weakness. Furthermore, heart palpitations, as well as impaired physical performance and cognitive functions, may occur. On the other hand, an overload of iron is equally dangerous. This element exhibits high chemical activity and tends to generate oxidative stress, which damages proteins, lipids, and DNA [7].

Iron occurs in food in two forms: heme iron, which comes from animal sources, and non-heme iron, which comes from plant sources. In populations that consume meat, heme iron accounts for only 10–15% of the total dietary intake of this element. However, due to its high and stable bioavailability, estimated at 15–35%, its role in the body is crucial. It can account for more than 40% of the total amount of absorbed iron [4,6]. In turn, legumes are characterized by a high mineral content, including iron; however, their bioavailability is limited. This is due to the presence of so-called anti-nutrients, which hinder the body’s full utilization of these valuable elements. Phytates and polyphenols present in these plants form insoluble complexes with minerals, thereby reducing their absorption. Ascorbic acid significantly increases the bioavailability of non-heme iron by reducing it to the absorbable Fe^2+^ form and creating soluble chelates that prevent its precipitation in the alkaline environment of the intestines [8,9]. The bioavailability of non-heme iron is significantly lower than that of the heme form found in animal products. By comparison, the estimated bioavailability of non-heme iron from dark green leafy vegetables is typically 7% to 9%. In cereals, this parameter drops to approximately 4%, while in legumes it is only about 2% [10,11]. Despite the widespread occurrence of iron in the environment and the relatively low daily requirement of the body, which amounts to approximately 1 mg of absorbed element from a 10 mg dietary intake, this nutrient remains one of the most common factors limiting the proper functioning of the organism [4]. Iron deficiency anemia remains a key challenge for contemporary public health. The epidemiological data indicate that this problem affects a significant percentage of the global population, particularly in groups with increased iron requirements, such as children (40%), pregnant women (37%), and women of reproductive and premenopausal age (30%) [12,13].

In recent years, there has been a significant increase in the popularity of dietary supplements on the market. By definition, their role is to supplement the daily diet, not to replace whole foods. Although in the case of diagnosed deficiencies, they can accelerate the restoration of homeostasis, it should be noted that, unlike medicinal products, supplements are not subject to such rigorous quality control. A study by Puścion-Jakubik et al. found that the majority of dietary supplements tested for iron content did not conform to the manufacturer’s declaration. Nearly 70% of the preparations tested contained a higher amount of iron than declared, and 11% were below the declared value. Less than 20% of the preparations met the declared amount of the element [14]. Other studies also confirmed the discrepancies in iron content in dietary supplements compared to the manufacturer’s declared content. Furthermore, these studies focused on quantitative determination of the element, taking into account the chemical and pharmaceutical forms, the conditions prevailing in the gastrointestinal tract, and the presence of other nutrients [15,16].

This study focuses on the evaluation of the relative bioaccessibility of iron supplied in the form of dietary supplements and medicinal products. The primary research objective was to determine the extent to which the type of diet, the chemical form of the element, and the formulation of the preparation affect the efficiency of their absorption by the body. To reflect real-world nutritional conditions, homogenates of total daily food rations were used in the research process. A two-stage in vitro digestion model was applied to simulate the processes occurring in the digestive system. An analysis of the resulting dialysate and post-digestive residue allowed for a precise determination of the relative bioaccessibility of iron. This approach made it possible to establish how the composition of the daily diet influences the efficiency of the release and absorption of the studied element from pharmaceutical preparations. For many years, research has been conducted on the concentration of elements in food and on estimating their actual intake using direct chemical analysis. The in vitro digestion method provides an important benchmark for obtaining reliable data on the bioaccessibility of mineral components from food products, including heavy metal contaminants, and its significant advantage is its high-time efficiency. Furthermore, conducting such laboratory analyses on food samples is procedurally simplified because, unlike studies involving human participants, it does not require approval from a bioethics committee [17,18]. Dietary supplements serve as the primary means for enhancing iron intake, and thus our main focus was on exploring the bioaccessibility of iron from these valuable and popular products. Additionally, we conducted a thorough assessment of the bioaccessibility of iron from two widely used iron-containing medications: one available over the counter (OTC) and the other accessible only by prescription. This investigation promises to shed light on the effectiveness of these sources in addressing iron deficiency.

## 2. Materials and Methods

### 2.1. Chemicals and Reagents

The in vitro digestion procedure used enzymes such as pancreatin (specific activity: 8xUSP), pepsin (specific activity ≥500 U/mg), and porcine bile extract, as well as 14 kDa molecular-cutoff cellulosic dialysis membranes purchased from Sigma-Aldrich (St. Louis, MO, USA). The sodium bicarbonate was purchased from Avantor Performance Materials (POCH, Gliwice, Poland). The high-purity deionized water (resistivity of 18.2 MW cm), obtained using an Ultrapure Millipore Direct Q-R 3UV (Millipore, Bedford, MA, USA), was used throughout the entire procedure. The suprapure grade nitric acid, hydrochloric acid, and hydrogen peroxide were from Merck (Darmstadt, Germany). The iron standard solutions were prepared using a 50 mg/mL stock solution (PlasmaCAL, SCP SCIENCE, Baie-D’Urfé, QC, Canada).

### 2.2. Materials

#### 2.2.1. Reconstruction of Diet Models

This study employed three diets commonly followed by healthy individuals: basic, standard, and high residue. The reconstruction of the daily food rations for each diet, comprising four meals (breakfast, mid-morning snack, lunch, and dinner), enabled the assessment of the effect of diet type on the bioaccessibility of iron from supplements and iron-containing medicinal products. All diets were prepared by a professional dietitian (A.J.) based on the current scientific literature [19,20], ensuring the optimal energy intake, as well as the appropriate macro- and micronutrient composition. The nutritional value of each diet was calculated using the Dieta 6.0 software (National Institute of Public Health–National Institute of Hygiene, Warsaw, Poland) and is presented in the Supplementary File (Table S1). A detailed quantitative and qualitative characterization of the diets is provided in the Supplementary File (Table S2).

Although the procedure for reconstructing daily food rations has been described in previous publications [21,22,23], a summary is included here. The diet reconstruction was conducted under laboratory conditions following the local dietary practices that were reported in the literature. All utensils were washed with detergent and hot water, rinsed with a diluted acid solution, and finally rinsed with high-purity deionized water. Ceramic, aluminum, and enamel utensils were excluded. Drinking water from the municipal supply in Lublin was used for food preparation, and the food products were sourced primarily from the local market, which was located in a major agricultural region. The spices were added in amounts consistent with local culinary practices.

The complete daily rations were homogenized using a homogenizer (Zelmer, Rzeszów, Poland) equipped with titanium blades and subsequently stored in plastic containers at −20 °C until analysis.

#### 2.2.2. Dietary Supplements and Medicinal Products

The study used eight iron-containing preparations purchased exclusively in pharmacies: six dietary supplements, one over-the-counter (OTC) product, and one prescription drug. The trade names and manufacturers were coded and presented numerically, as the study’s purpose was not to evaluate the quality of particular products. The selection of the products was based on the knowledge and experience of the certified pharmacists (P.B., W.K., and J.T.) who hold an active license to practice in Polish pharmacies. The products were selected primarily based on consumer popularity, with additional criteria including wide pharmacy availability and long expiration dates. The selection also aimed to ensure diversity in the chemical and pharmaceutical forms of iron. The detailed product characteristics are provided in Table 1. Additionally, Table S3 in the Supplementary File provides a detailed composition of each product.

Each product was obtained from three different batches, and three analytical samples were performed for each batch. The bioaccessibility of the iron was determined in 27 different experimental models (24 models using dietary supplements or medicinal products and 3 models for diets alone). Considering the number of dietary supplements and drugs, the types of diets used, and the number of replicates, a total of 228 samples were analyzed (24 models × 3 different batches × 3 replicates and 3 types of diets × 3 replicates and 3 control samples). Considering that, according to the procedure used in the current study, after each in vitro digestion, two fractions were obtained, and the concentration of iron was determined in a total number of 456 analytical samples. Additionally, the iron concentration was determined in 6 samples of the reference material. Therefore, a total number of 462 analytical samples was subjected to ICP-OES determinations.

### 2.3. Enzymatic Two-Phase In Vitro Digestion Model

In the present study, a static gastrointestinal in vitro digestion model consistent with the INFOGEST protocol was applied. The oral phase was omitted, and food grinding was replaced by prior homogenization of the food rations, which is acceptable within the INFOGEST framework [24]. Subsequently, the gastric and intestinal digestion phases were conducted under conditions closely resembling the physiological ones, using appropriate enzymes, temperature, and mixing of the food contents. The procedure was based on the in vitro digestion model originally proposed by Miller [25] and was modified by incorporating semipermeable dialysis membranes. This model has been previously validated for assessing the bioavailability of minerals and bioactive food compounds [21,22,23,26,27].

The first stage of in vitro digestion corresponded to the gastric phase. Briefly, 25 g of dietary homogenate was transferred into polypropylene containers and diluted to a final mass of 50 g with high-purity deionized water. This setup was used to determine the iron bioaccessibility from the diet alone. To assess the iron bioaccessibility from dietary supplements and medicinal products under the influence of diet, one dose of the pharmaceutical product was added to 25 g of dietary homogenate before being diluted to 50 g. The pH was adjusted to 2.0 using 2 mol/L HCl, and 2 mL of 10% (*w*/*v*) pepsin solution prepared in 0.1 mol/L HCl was added. The samples were tightly sealed and incubated in a thermostatic shaking water bath at 37 °C for 2 h.

Following the gastric phase, the pH of the digest was adjusted from 2.0 to 6.5 using a 6% NaHCO_3_ solution. The intestinal phase was initiated by the addition of 5 mL of a 0.4% pancreatin solution prepared in 0.1 mol/L NaHCO_3_. To simulate intestinal absorption, the digest was transferred into cellulose dialysis membranes that were previously soaked for 12 h in 0.1 mol/L HCl and thoroughly rinsed with high-purity deionized water. The filled membranes were sealed with dedicated clips and placed in polypropylene containers containing 500 mL of high-purity deionized water. The samples were incubated in a thermostatic shaking water bath at 37 °C for an additional 2 h. A blank control, prepared without dietary homogenate or pharmaceutical product, was subjected to the same digestion procedure.

**Figure 1:**
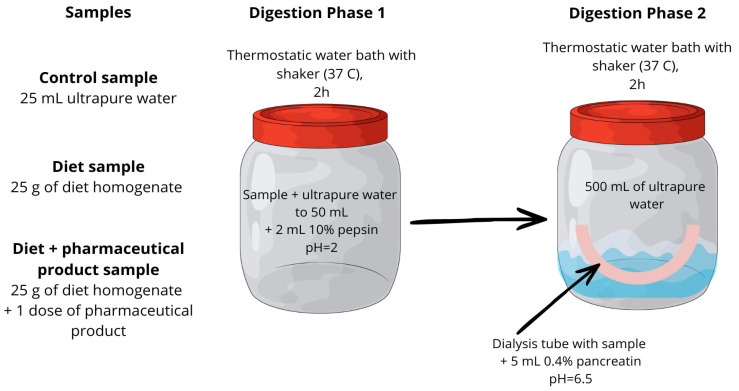
A graphical summary of the applied in vitro digestion procedure.

At the end of the in vitro digestion, two fractions were obtained: the dialyzable fraction (dialysate) and the non-dialyzable fraction retained within the dialysis membrane. Both fractions were collected and subjected to further analysis. A graphical illustration of the experimental model was presented in Figure 1.

### 2.4. Digestion of Samples

The samples obtained after the in vitro digestion step were subjected to open-vessel acid digestion with condensate recirculation using a DigiPREP MS heating block (50 mL, 48-position) equipped with a DigiVAC extractor and a DigiPREP KeyPad controller (SCP SCIENCE, Baie-D’Urfé, QC, Canada).

- Digestion of the dialyzable fraction

Here, 5 mL of the dialysate was transferred into a DigiTUBE vessel (SCP SCIENCE, Canada), and 1 mL of the 65% (*v*/*v*) HNO_3_ was added. The samples were digested for 2 h at 120 °C. After cooling, the digests were filtered through DigiFilters (SCP SCIENCE, Canada) using a Rocker 300 vacuum pump (Rocker Scientific, New Taipei City, Taiwan) and diluted to a final volume of 10 mL with high-purity deionized water.

- Digestion of the nondialyzable fraction

Here, 1 g of the dialysis membrane residue was placed in a DigiTUBE vessel, and 3 mL of the 65% (*v*/*v*) HNO_3_ was added. The samples were left to pre-digest for 24 h at room temperature. Subsequently, 1 mL of the 30% (*v*/*v*) H_2_O_2_ was added, and mineralization was performed for 2 h at 120 °C. After cooling, the digests were filtered through DigiFilters using a Rocker 300 vacuum pump and diluted to a final volume of 10 mL with high-purity deionized water.

### 2.5. Analytical Determinations of Iron Using Inductively Coupled Plasma Optical Emission Spectrometry (ICP-OES)

Each digest solution was analyzed in triplicate by inductively coupled plasma optical emission spectrometry (ICP-OES) using a high-resolution PlasmaQuant 9000 Elite spectrometer (Analytik Jena, Jena, Germany). The instrument operating parameters were provided in Table S4 in the Supplementary Material. The equipment calibration was performed using appropriate dilutions of a certified standard Iron PlasmaCAL solution (50 mg/mL).

The analytical method had been previously validated for the determination of iron, Cr, Se, and Mg [28,29]. However, because a different mineralization procedure was applied, the validation was repeated using a reference material consisting of a flour–milk mixture (7:3, m/m), which was enriched with known amounts of the analyzed elements. This reference material was analyzed six times following the same procedure used for the test samples. The results were summarized in Table 2.

### 2.6. Calculation of the Bioaccessibility Value

Based on the iron content in the dialysates and the residues in the dialysis membranes, the bioaccessibility (expressed as a percentage) was calculated according to the following formula:

$$B\%=\frac{D+Dr}{T+D}\times 100\%$$

where

B%—the relative bioaccessibility of iron;

D—the amount of Zn (mg) in the dialysate;

T—the amount of Zn (mg) in the digest of the dialysis tube residue;

Dr—the amount of iron (mg) corresponding to the equilibrium of concentrations on both sides of the semipermeable membrane, which were present inside the dialysis tube.

The Dr was calculated using the following equation:

$$Dr=\frac{\left(Cd-Cc\right)\times Vt\times R}{1000}$$

where

Cd—the concentration of iron in the dialysate solution (μg/mL);

Cc—the concentration of iron in the control sample (μg/mL);

Vt—the volume of the dialysis tube (mL);

R—the dilution factor.

The bioaccessibility of iron was assessed using four research models: from the diet alone; from the pharmaceutical products in the presence of a diet; and as a function of the chemical form of iron and the pharmaceutical formulation.

### 2.7. Statistical Analysis

The bioaccessibility data were compiled using Microsoft Excel, and the statistical analyses were performed with Statistica v. 13.0 (StatSoft, Kraków, Poland). The descriptive statistics, including the arithmetic mean (x, M), median (Me), standard deviation (SD), minimum (Min), maximum (Max), interquartile range (IQR), as well as the F and H values of the ANOVA test statistics, were used to summarize the results. The Shapiro–Wilk test was applied to assess the normality of the data distribution. The parametric tests were used when the normal distribution was confirmed, whereas the nonparametric tests were applied when the assumption of normality was not met.

To evaluate the differences in iron bioaccessibility among the pharmaceutical products, the one-way analysis of variance (ANOVA) followed by Tukey’s post hoc test was conducted. The effects of the chemical forms of iron and the pharmaceutical dosage forms on relative bioaccessibility were analyzed using one-way ANOVA with Tukey’s post hoc test or, when appropriate, the Kruskal–Wallis rank-based ANOVA with Dunn’s post hoc test. For all the statistical analyses, the level of significance was set at α = 0.05. The statistical significance was interpreted as follows: *p* < 0.05 indicated statistical significance, *p* < 0.01 strong statistical significance, and *p* < 0.001 very strong statistical significance.

## 3. Results

### 3.1. Bioaccessibility of Iron Under the Influence of Various Diets

Table 3 presents the bioaccessibility results for iron from selected dietary supplements and medicinal products in the context of various dietary patterns. The data obtained for the diet alone, without the addition of supplements, provides a reference point illustrating the natural bioaccessibility of this element resulting solely from the composition of meals. The comparative analysis showed that the type of diet used and the choice of medicinal product or dietary supplement significantly determine the relative bioaccessibility of iron. In the experiments conducted without the addition of external preparations, the relative bioaccessibility of iron ranged from 4.63% to 7.96%. The highest value was determined for the basic diet (7.96%), which proved significantly more effective than the high-residue diet (*p* < 0.05) and the standard diet (*p* < 0.001). The lowest bioaccessibility was demonstrated for the standard diet (4.63%). When examining the relative bioaccessibility of iron from diets using selected dietary supplements or medicinal products, its value within individual preparations varied. For products one, three, and five, the bioaccessibility of iron from the standard diet was statistically significantly lower compared to the basic diet (*p* < 0.001) and the high-residue diet (*p* < 0.001). In contrast, an inverse correlation was observed for products four, six, and eight. In these models, the standard diet achieved significantly higher iron bioaccessibility compared to the other dietary models. Furthermore, the analysis showed that product No. eight had the highest bioaccessibility of all tested products for each dietary model, and it achieved the highest bioaccessibility when combined with the standard diet (21.1%). Interestingly, in the analyzed research models, the basic diet provided the optimal environment for iron bioaccessibility from most supplements compared to the other diets. Although the highest bioaccessibility rates were observed for most of the tested supplements, these differences were not always statistically significant. The mean values of relative iron bioaccessibility in the models based on the basic diet ranged from 3.65% to 18.59%, with the highest effectiveness demonstrated for product No. eight and the lowest for supplement No. five. The lowest values of relative iron bioaccessibility were obtained in models using dietary supplement No. five (1.92–3.65%).

It is worth noting that for product No. seven, the current study did not reveal any significant differences in iron bioaccessibility between the standard and high-residue diets. It should also be emphasized that none of the tested products found the bioaccessibility of iron to be the highest in the presence of a high-residue diet, although the differences in comparison to the other two types of diets were not always significant.

### 3.2. Influence of Diet and Chemical Form on Iron Bioaccessibility

The pharmaceutical products evaluated in this study contained iron in the form of fumarate, lactate, sulfate, gluconate, and an amino acid chelate (bisglycinate). Each chemical form was assessed for its effect on iron’s relative bioaccessibility across the dietary models employed, with the corresponding results summarized in Table 4. The average values for the particular iron salts obtained from the tested products are presented in Table 5.

For iron (II) fumarate, bioaccessibility was highest under the influence of a basic diet (9.19%) and differed significantly from that observed for the other two diets. In addition, the bioaccessibility of iron in the presence of a standard diet (3.06%) was significantly lower than that observed under the influence of a high-residue diet (5.83%). For iron (II) lactate, the bioaccessibility values ranged from 4.56 to 6.02, with the highest value observed for the basic diet model. No significant differences were found between the standard and high-residue diets. The bioaccessibility of iron (II) sulfate ranged from 11.67 to 14.02%, with no significant differences observed among the three dietary models. For iron bisglycinate, the highest results were observed in the presence of the standard diet (9.77%) and differed significantly from those of the other two dietary models. No significant differences were found between the basic (6.25%) and high-residue (5.92%) diets. No significant differences in iron bioaccessibility from gluconate were observed between the standard and high-residue diets. However, the highest results were obtained in the model using the basic diet (10.0%), which was significant in comparison to the two other diets used in the study.

According to the data presented in Table 5, on average (for all dietary models), iron sulfate (12.58%) was characterized by the highest iron bioaccessibility, significantly higher than that observed for fumarate (6.03%), lactate (5.25%), and the diet alone (6.36%). No significant differences in diet-related bioaccessibility were observed between gluconate (8.92%) and bisglycinate (7.32%). However, fumarate showed a lower iron bioaccessibility compared with bisglycinate and gluconate. Similar findings were observed for lactate, which demonstrated a lower bioaccessibility than gluconate and bisglycinate.

### 3.3. Influence of the Pharmaceutical Form on the Bioaccessibility of Iron

The dietary supplements and medicinal products containing iron compounds included in the study were available in four pharmaceutical forms: coated tablets, extended-release tablets, capsules, and tablets. The evaluation of the effect of pharmaceutical form on iron bioaccessibility demonstrated, as shown in Table 6, that the extended-release tablets were characterized by the highest bioaccessibility values, which were significantly higher in comparison to other formulations. Conversely, conventional tablets exhibited the lowest bioaccessibility, with values significantly lower than those obtained for coated tablets and capsules. No significant differences were identified between capsules and coated tablets.

## 4. Discussion

Iron deficiency is the most prevalent mineral deficiency worldwide. According to the World Health Organization, approximately 50% of anemia cases are attributed to insufficient iron levels in the body, primarily due to inadequate dietary intake [30]. Pharmaceutical products, including dietary supplements, are commonly used to correct these deficiencies. However, the iron content in such preparations alone does not accurately reflect their effectiveness in supplementation; rather, the bioaccessibility is a critical factor, influenced by multiple variables.

The relative bioaccessibility of iron in the analyzed dietary systems, without the addition of pharmaceuticals or dietary supplements, ranged between 4.63% and 7.96%. The highest level of bioaccessibility for this element was determined for the basic diet (7.96%), while the lowest was found for the standard diet (4.63%). The standard diet was characterized by significantly lower iron bioaccessibility compared to both the basic and the high-residue diets (*p* < 0.01). The basic diet provided significantly higher iron absorption values in comparison to the high-residue diet. The obtained results may suggest an influence of Ca content on the limitation of iron availability. The basic diet was characterized by the lowest Ca content, whereas the standard and high-residue diet models contained more than twice the Ca content of the basic diet. The studies have shown that calcium has a negative impact on the absorption of both non-heme and heme iron [31,32,33]. In a systematic review and meta-analysis of randomized and crossover trials, a statistically significant reduction in iron bioavailability due to Ca was demonstrated in the short-term studies (≤90 days) [34]. The significantly lower iron bioaccessibility from the standard diet also correlates with the lowest vitamin C content among the analyzed dietary models. In the case of the high-residue diet, despite a high intake of fiber, which may limit the absorption of iron, an almost 5-fold higher concentration of ascorbic acid was recorded compared to the standard diet. This represents a key factor modulating the final iron bioaccessibility. Many studies indicated the beneficial effect of vitamin C on the availability of iron from food [35,36]. Interestingly, the results of randomized trials by Li N. et al. suggested that vitamin C supplements are not essential when taken alongside oral iron supplements for patients with anemia [37]. An additional factor limiting iron absorption may be the polyphenols contained in beverages such as coffee or tea. According to current knowledge, these compounds significantly limit the absorption of iron from the daily diet, especially if the time between a meal or a beverage containing polyphenols is not maintained [32,38,39,40]. The results obtained in this study confirmed that iron absorption from the standard diet, which was dominated by polyphenol-rich beverages, was the lowest compared to other types of diet. It was also observed that, under a standard diet, the lowest bioaccessibility was associated with supplement No. five (1.92%). This supplement contained the highest level of polyphenolic compounds among all those tested, as well as 107.48 mg of caffeine, which was absent from the other supplements. Similar trends were observed under the high-fiber diet conditions. Products one and three, both containing iron fumarate, exhibited a bioaccessibility of 6.63% and 7.36%, respectively, whereas formulation five showed a markedly lower bioaccessibility of 3.50%. Comparable relationships were also noted for the basic diet model. These findings suggest that the polyphenolic compounds present in formulation five may have played a key role in reducing the bioaccessibility. On the other hand, the lower iron bioaccessibility that was observed in products one and five, compared with the other preparations, may be attributed to the excipients used. Both products contained calcium phosphate, which may promote the formation of insoluble iron phosphate. According to Baumgartner et al., this compound has limited nutritional value in humans due to its poor absorption [41]. Although iron phosphate is relatively stable in food matrices, its bioavailability is approximately half that of iron sulfate. Notably, only nanostructured forms of this salt have been shown to enhance bioavailability, suggesting their potential applicability in food fortification and dietary supplementation [42].

A study using an in vitro digestion model has demonstrated the effect of phytic acid on limiting iron absorption. The phytase-supplemented sample showed less than half the available iron content (49%) compared to the reference sample (Ref. = 100%). It has also been suggested that dietary protein may play a significant role in iron binding [17]. The results of the current study are not fully in agreement with these observations, pointing to the potential role of phytic acid from a high-residue diet as a factor limiting iron bioaccessibility. However, in the present study, the high-residue diet was characterized by a lower protein content compared to the standard diet, which demonstrated the lowest iron bioaccessibility. This indicates many interactions in complex food matrices, where it is difficult or impossible to identify one main decisive factor.

The results of studies assessing the bioaccessibility of iron from various food products using a simulated in vitro digestion model are highly variable. Bryszewska, examining the bioaccessibility of iron from dietary supplements, demonstrated a reduction in bioaccessibility in the presence of a food matrix for iron sulfate and lactate (10–28%, depending on the research model used), although the differences were not significant [43]. This study also demonstrated a much lower bioaccessibility of iron after only the gastric phase (40–90%), compared to results assessed after full gastrointestinal digestion (8–30%). This indicates the limiting effect of the alkaline environment on the bioaccessibility of iron^2+^ ions. A beneficial effect of vitamin C on iron bioaccessibility was also demonstrated in the full gastrointestinal model, which is consistent with the results obtained in this study. In a similar study using an in vitro digestion procedure with cellulose dialysis membranes and ICP-MS determinations, Sajkowska et al. [44] revealed the average bioaccessibility of iron from commercially used herbs (basil, peppermint and rosemary) at the level of <2%, which proved to be a very poor availability for the body of this trace element from plant-derived products. Saliburska et al., in turn, found the highest bioaccessible iron fraction after an in vitro digestion of cashew nuts (51.1%) and green lentils (32.6%). Relatively high values were also obtained for corn (32.1%) and white rice (21.4%) [45]. Kapsokefalou et al. [46] conducted a study using an in vitro digestion model combined with dialysis to evaluate the iron bioaccessibility from pasteurized milk enriched with vitamin C and various iron salts. The dialyzability of iron from bisglycinate, lactate, and pyrophosphate was 5.7 ± 3%, 7.1 ± 3.7%, and 8.2 ± 5.1%, respectively, with no significant differences observed among these salts. In contrast, lower dialyzability values were reported for sulfate and gluconate, at 1.9 ± 1.2% and 4.2 ± 3%, respectively. Using a similar model, Jaiswal et al. [47] determined that the bioaccessibility of iron from a complementary mixed meal (consisting of, among others, wheat flour, soy flour, and milk) supplemented with ferrous fumarate was 15.7% and was 17.2% from NaFeEDTA, but these differences were considered insignificant. Importantly, the meals studied were phytate-reduced. A two-fold reduction in the molar ratio of phytates to iron increases the bioaccessibility of this mineral [47]. The studies by Kloots et al. [48] also showed that the bioaccessibility of iron from fortified cereal products strongly depends on the chemical form of the iron compound. They reported that the most commonly used iron salts, including ferrous sulfate, ferrous fumarate, ferrous lactate, and ferric pyrophosphate, had a very low dialyzable iron content after in vitro digestion, whereas NaFeEDTA showed a significantly higher bioavailability in the Caco-2 cell model. The dialyzability value of iron for NaFeEDTA was 5.7%, while for the other salts it ranged from 0.6% to 0.9% [48].

Dietary supplements may contain both non-heme and heme forms of iron. Iron (II) salts, such as sulfate, fumarate, and gluconate, exhibit a bioaccessibility of approximately 10–15%, while iron (III) salts show 3–4 times lower bioaccessibility [49]. The present study showed that preparations containing iron in the form of ferrous sulfate exhibited the highest degree of relative iron bioaccessibility (12.58%). The high bioaccessibility of this chemical form of iron may result from its ability to dissociate rapidly in the acidic environment of gastric juice. The released iron^2+^ ions constitute a direct substrate for the divalent metal transporter located in the brush border of the duodenal enterocytes [50]. In the current study, relatively high results of iron bioaccessibility were also obtained in the case of preparations containing the chemical forms of ferrous gluconate (8.92%) and iron bisglycinate (7.31%). In turn, Falahati et al., in a study involving 120 healthy children, found that although both iron sulfate and iron gluconate were effective in the prevention of anemia, the group receiving iron gluconate exhibited higher hemoglobin (12.11 g/dL) and ferritin (61.00 µg/L) levels than the group receiving iron sulfate (Hb level 11.85 g/dL; ferritin level 58.25 µg/L) [51]. In a study of school-age children receiving iron sulfate or iron bisglycinate, Duque et al. reported that supplementation with both forms was similarly effective. However, higher ferritin levels were observed in the bisglycinate group (31.0 µg/L) compared to the sulfate group (25.2 µg/L) after six months of supplementation. This difference was no longer evident at the end of the study period (nine months) (29.6 vs. 28.9 µg/L). The hemoglobin levels did not change significantly in either group throughout the supplementation period [52]. Additionally, the study by Santiago et al. [53], which was conducted in a cohort of pregnant women aged 18–40 years, did not demonstrate significant differences in the mean hematological parameters between the groups receiving iron bisglycinate and iron sulfate. However, the amino acid chelate was associated with greater bioavailability at a lower dose and improved tolerability [53]. Similar conclusions were obtained by Milman et al., and bisglycinate was better tolerated than sulfate [54]. Another study revealed that iron (II) fumarate has a bioavailability and gastrointestinal tolerability profile similar to iron (II) sulfate in healthy individuals [55]. The present study demonstrated a significantly lower bioaccessibility of iron fumarate compared with iron bisglycinate. However, in an observational clinical study, Suva et al. reported no significant differences in hemoglobin levels between the fumarate (11.72 g/dL) and bisglycinate groups (11.69 g/dL) following supplementation [56]. Similarly, in the present study, it was demonstrated that iron gluconate was characterized by significantly higher bioaccessibility than iron fumarate. In contrast, Lin et al., in an in vitro study using Caco-2 cells, reported no significant differences in iron bioaccessibility between gluconate- and fumarate-enriched rice flours in either low- or high-phytic acid groups. However, an 11.53–13.45% higher iron absorption rate was observed from the gluconate-enriched flour compared with the fumarate-enriched flour [57]. An interesting point of reference is the in vitro research conducted by Zhu et al. using the Caco-2 cell line. The authors demonstrated that under the specific conditions of the cellular model, pyrophosphate salts may exhibit a higher bioaccessibility than chelated forms, sulfates, or iron chlorides. It was also shown that a change in pH from 2 to 7 resulted in a decrease in bioavailability by 40.6, 72.6, and 13.4% for soluble ferric pyrophosphate (SFP), FeSO_4_, and NaFeEDTA, respectively, which indicates that iron from FeSO_4_ is more sensitive to pH changes than chelated forms (SFP, NaFeEDTA) [58]. This suggests that the final absorption efficiency of the element is a result of not only the chemical form of the compound but also the specificity of the matrix and the conditions prevailing in the intestinal environment.

The results obtained in the present study suggest that, in addition to the chemical form of iron and dietary factors, the pharmaceutical formulation of a preparation may influence the iron bioaccessibility. For iron supplementation, pharmaceutical formulation characteristics appear particularly relevant, as they may contribute to reducing gastrointestinal adverse effects, which are a common reason for treatment discontinuation or irregular supplement use [59,60].

The present findings indicate that the extended-release formulation was associated with the highest determined bioaccessibility (19.31%). This preparation was a registered medicinal product meeting the established regulatory quality standards, whereas dietary supplements are not required to fulfill equivalent criteria. While the higher elemental iron content (105 mg) of this formulation may have contributed to the observed bioaccessibility, the results likely reflect the combined influence of multiple formulation-related factors rather than dosage alone. Furthermore, a review by Sharawat et al. reported that iron supplementation at a dose of 60 mg was more effective than 30 mg, while no additional benefit was observed with doses exceeding 60 mg for the prevention of anemia in pregnant women [61]. The previous literature suggests that extended-release iron preparations may offer advantages over immediate-release formulations in terms of absorption dynamics and tolerability [49]. The extended-release (SR/XR) formulations are thought to allow iron to reach the duodenum with a reduced premature release, enabling a gradual delivery along the small intestine and potentially supporting a sustained absorption. In standard and immediate-release formulations, iron is primarily released in the stomach, after which it enters the more alkaline intestinal environment, where it may undergo oxidation to the less readily absorbable ferric form [49,59]. On the other hand, at the top of the enterocytes, duodenal cytochrome B (Dcyt B) uses vitamin C to reduce Fe^3+^ to Fe^2+^ [62]. Additionally, the conventional formulations may leave greater residual iron in the gastrointestinal tract, which could contribute to a higher incidence of adverse effects [63]. Moreover, excess unabsorbed iron progresses to the large intestine, where it may induce intestinal dysbiosis characterized by the proliferation of pathogenic microorganisms and a concurrent reduction in beneficial microbial populations [64]. Controlled-release preparations have also been proposed to reduce the likelihood of interactions with concomitant medications, such as proton pump inhibitors, although the clinical significance of this effect may vary [59].

In contrast, Kaltwasser et al., using the stable isotope ^54^Fe in 18 healthy adult men following phlebotomy, reported no significant difference in iron absorption between a rapid-release formulation containing ferrous ascorbate (150 mg Fe^2+^) and a sustained-release formulation containing ferrous sulfate (160 mg Fe^2+)^. The average iron utilization from both forms was virtually identical for both preparations (23 and 22%, respectively). [65]. Zariwala et al., based on the in vitro studies using Caco-2 cells, demonstrated that iron absorption from a conventional-release ferrous sulfate tablet was greater than that from a modified-release formulation. Iron absorption from conventional-release iron (II) sulfate tablets was 41% of the FeSO_4_ standard. In contrast, for ferrous fumarate, no significant differences in absorption were observed between the conventional and delayed-release tablet formulations [66].

In the present study, capsules and coated tablets demonstrated higher bioaccessibility than uncoated tablets. Navarro et al. reported lower absorption from crushed tablets or powdered supplements, potentially due to increased interactions with dietary components, whereas maintaining the intact tablet form may limit such interactions. The difference in the 12 h area under the curve (AUC) compared to the control group for crushed tablets was 7%, while in the case of the supplement in the form of whole tablets, the AUC was 19% higher compared to the control group [67]. Similar conclusions were drawn by Hartman-Craven et al. The relative bioavailability (measured by AUC) for the iron powder supplement was 10 ± 43.3 µmol·h/L, and for the iron tablets it was 41.8 ± 45.9 µmol·h/L [68]. The coating may additionally delay the tablet disintegration [69] and mask the bitter taste, potentially enhancing adherence, particularly in pediatric populations [70]. However, the available evidence remains inconsistent. Latif et al., in an in vitro model, reported that the bioaccessible fraction from the coated tablets containing iron was below 25%, whereas for both hard and soft capsules, the bioaccessible fraction did not reach the detectable limit [71]. In contrast, Thankachan et al. [72], in a study of nine healthy women aged 18–40 years, demonstrated a greater iron absorption from the ^58^Fe-labeled uncoated tablets compared with the ^57^Fe-labeled enteric-coated tablets (12% vs. 5%). The reduced absorption observed with enteric-coated tablets may be attributable to the limited iron release in the duodenum, which is the primary site of iron absorption, potentially due to shortened duodenal transit time [72]. Overall, the effect of pharmaceutical form on iron bioavailability requires further investigation in well-designed in vivo studies.

Although a two-stage in vitro digestion model was applied under conditions consistent with the INFOGEST protocol (i.e., enzyme composition, temperature, and mixing conditions), several inherent methodological limitations may have influenced the obtained results. The applied model represents a useful tool for estimating iron bioaccessibility and for assessing the influence of dietary factors on iron release. Some evidence suggests a reasonable correlation between the in vitro and in vivo findings [73,74]. However, such models do not reflect actual bioavailability. Therefore, the term bioaccessibility is more appropriate, as it refers only to the fraction of iron released from the food matrix, remaining soluble and potentially available for intestinal absorption. An accurate assessment of bioavailability requires costly, time-consuming, and ethically regulated in vivo studies that account for the complex physiological factors. Comparing the bioavailability/bioaccessibility results is difficult due to the different research models used in many studies. This primarily reflects the differences between the results obtained using in vivo and in vitro methods, but also those from in vitro studies using cell lines, studies of iron release from the food matrix, or more advanced methods using dialysis tubes, which allow for the separation of the element from high-molecular-weight compounds and for the analysis of only the dialyzable fraction with potentially high bioavailability. To the best of our knowledge, there is no data in scientific journals on the effect of diet type on the bioaccessibility of iron from pharmaceutical products; therefore, this study provides new, interesting information on the effect of a complex food matrix on the bioaccessibility of iron from dietary supplements and medicinal products containing various chemical forms of the element. A major limitation of the in vitro models employing dialysis membranes concerns the simulation of iron transport. The dialysis approach accounts exclusively for passive diffusion, which plays a negligible role in iron absorption. In vivo non-heme iron uptake is mediated primarily by divalent metal transporter 1 (DMT1), whereas heme iron absorption occurs via distinct transport mechanisms that remain incompletely characterized. Moreover, systemic iron absorption is tightly regulated by hepcidin, a hepatic peptide hormone that decreases the iron uptake under conditions of elevated body iron stores. The recent evidence indicates that alternate-day iron supplementation enhances fractional iron absorption, likely due to reduced hepcidin levels following the dosing intervals [75,76]. Additionally, intestinal iron transporters are locally regulated by a hypoxia-inducible factor (HIF)-2α in enterocytes [61]. These regulatory mechanisms cannot be replicated in static in vitro systems. Moreover, a major limitation of the dialysis method is that the rapid increase in pH during intestinal digestion renders a significant fraction of insoluble iron, thereby reducing its bioaccessibility and potentially affecting the results [77]. Another limitation is the exclusion of the large intestine phase in the applied digestion model. Cai et al. demonstrated that iron bioaccessibility in the colon may be 1.3–1.8-fold higher than in the small intestine, suggesting a potential contribution of gut microbiota to iron absorption. The anaerobic environment and reducing activity of colonic bacteria facilitate the conversion of Fe^3+^ to the more soluble Fe^2+^ form, which may enhance absorption [78]. Consequently, iron bioaccessibility may be underestimated in the present model. Furthermore, all the tested preparations were administered under identical simulated digestive conditions. In real-life settings, iron supplements are consumed with or without food, in the fasting state, or alongside specific meal compositions, all of which may significantly affect absorption. Although investigating these variables would require an impractically large number of experimental conditions, future studies should consider a more diverse and representative set of formulations to better compare the pharmaceutical forms. The influence of dose on iron absorption was also not assessed. This is particularly relevant for dietary supplements, for which the manufacturer-declared iron content should ideally be analytically verified. Numerous studies have demonstrated that dietary supplements frequently fail to comply with declared ingredient specifications [14,79]. Puścion-Jakubik et al. reported that only 19.27% of the 109 iron preparations that they analyzed contained iron in amounts consistent with the manufacturer’s declared content [14]. The main goal of the research was to best reflect the actual conditions in which iron supplements or medications are taken; therefore, in the research model, we calculated the bioaccessibility based on the recommended intake portion, not the specific iron content or its concentration.

Nevertheless, the present study has several strengths. The iron bioaccessibility was determined using a validated analytical method combined with appropriate statistical analysis. A relatively large number of preparations were included, representing different chemical compounds and pharmaceutical forms, analyzed in multiple series with triplicate measurements for each series. Moreover, the impact of three distinct dietary models was evaluated, providing comparative data that are scarce in the current literature. Another strong side of the present study is that we have assessed the bioaccessibility of iron from whole, intact pharmaceutical forms (as swallowed) because tablet crushing, as observed in many publications, significantly impacts the potential absorption of the ingredients. It is important to remember that the tablets or capsules were swallowed whole and were not chewed.

Despite the methodological limitations described above, primarily related to the active transport of iron in an in vivo model, the literature contains numerous studies on the potential bioaccessibility of iron in a simulated in vitro digestion model. The results of the selected studies based on a similar research model were presented above. It is important to properly understand the results of such studies. It is important to emphasize that the terms bioavailability and bioaccessibility are not the same and should not be used interchangeably [80]. The term bioavailability refers to the fraction of a substance that enters the bloodstream and can be utilized by the body. Obtaining such results is only possible in an appropriate in vivo model. Such studies are time-consuming, require approval from relevant bioethics committees, and, in the case of human studies, are not possible for toxic substances (e.g., toxic heavy metals) [81,82]. The term bioaccessibility, in turn, refers only to the potentially bioavailable fraction that can be absorbed by the body after being released from the food matrix [83,84] and is only one of the three main factors influencing oral bioavailability, in addition to absorption and transport [85,86,87]. Therefore, in this case, the absorption mechanism itself remains a secondary issue, as this model does not assess direct absorption but only the fraction potentially available for absorption. The advantages of this type of study include simplicity, rapidity of execution, lack of ethical issues, and the ability to assess toxic substances, as living organisms are not exposed to their potentially harmful effects. This is why, in recent years, numerous in vitro studies have appeared in the scientific literature assessing the bioaccessibility of various nutrients using several in vitro models [24,44,88,89]. Furthermore, such models allow for extensive modifications of the research model, which was performed in this study, where the assessed supplements and medicinal products were mixed with various diets to evaluate the effect of dietary composition on iron bioaccessibility.

## 5. Conclusions

The study demonstrated that iron bioaccessibility is influenced by diet composition, its chemical form, and the pharmaceutical formulation of the supplement or drug. Although dietary fiber may negatively affect iron bioaccessibility, this hypothesis was not fully confirmed. This may be associated with the high vitamin C content of the high-residue diet, which also exhibited the highest fiber levels. In the absence of pharmaceutical supplementation, the highest iron bioaccessibility was observed with the basic diet. Among the tested formulations, iron(II) sulfate showed the highest bioaccessibility, with extended-release tablets proving to be the most effective. These findings indicate that iron bioaccessibility is determined by multiple factors. The present study revealed that an in vitro digestion model combined with ICP-OES analysis and appropriate statistical methods constitutes a useful tool for evaluating the non-human factors affecting this process. Although this approach has limitations, it offers a cost-effective and rapid alternative that enables preliminary observations, screening studies, and hypothesis generation without ethical constraints.

## Figures and Tables

**Table 1:** The detailed specification of the products containing iron used in the study.

Product No	Chemical Form	Iron Content/Iron Dose [mg]	Pharmaceutical Form	Product Type
1	iron fumarate	14	coated tablet	vitamin–mineral with plant extracts dietary supplement
2	iron lactate	9.9	coated tablet	vitamin–mineral with plant extracts dietary supplement
3	iron fumarate	14	tablet	vitamin–mineral dietary supplement
4	iron sulfate	9.9	coated tablet	vitamin–mineral dietary supplement
5	iron fumarate	12	tablet	vitamin–mineral with plant extracts dietary supplement
6	iron bisglycinate—Ferrochel^®^ TRAACS^®^ (iron amino acid chelate)	14	capsule	single mineral dietary supplement
7	iron gluconate	23.2	coated tablet	OTC drug
8	iron sulfate	105	extended-release tablet	prescription drug

**Table 2:** The analytical method validation results.

Parameter	Iron (ICP-OES)
Reference value (mg/kg)	22.9
Determined value (mg/kg)	20.1
	22.4
	23.9
	19.8
	22.5
	21.9
Average	21.8
SD	1.56
RSD (%)	7.16
Recovery (%)	95.2
LOD (µg/kg)	161.0
LOQ (µg/kg)	536.3

Abbreviations: SD—standard deviation; RSD—relative standard deviation; LOD—limit of detection; and LOQ—limit of quantification.

**Table 3:** The bioaccessibility of iron from dietary supplements and medicines under the influence of various types of diets.

Dietary Supplement No.	Chemical Form	Diet	M	Me	Min	Max	IQR	SD	One-Way ANOVA		Tukey’s Post Hoc Test Results		
									F	*p*	Group 1	Group 2	*p*
Without (%)	-	Standard	4.63	4.68	3.31	5.89	0.72	0.76	26.35	<0.001	Standard	Basic	<0.001
		Basic	7.96	8.39	5.93	9.27	1.21	1.04			Standard	High-residue	<0.01
		High-residue	6.48	6.74	4.86	7.84	1.24	1.10			Basic	High-residue	<0.05
1	iron fumarate	Standard	2.94	2.96	1.51	4.38	0.38	0.76	127.59	<0.001	Standard	Basic	<0.001
		Basic	7.55	7.39	6.87	8.45	0.81	0.56			Standard	High-residue	<0.001
		High-residue	6.63	6.68	5.66	7.60	0.53	0.61			Basic	High-residue	>0.05
2	iron lactate	Standard	4.56	4.62	3.92	5.13	0.75	0.42	9.30	<0.001	Standard	Basic	<0.001
		Basic	6.02	6.34	4.20	7.84	1.40	1.10			Standard	High-residue	>0.05
		High-residue	5.16	5.28	4.55	5.76	0.61	0.43			Basic	High-residue	>0.05
3	iron fumarate	Standard	4.32	4.33	2.75	6.41	1.22	1.19	398.84	<0.001	Standard	Basic	<0.001
		Basic	16.37	16.84	14.95	17.58	1.57	1.06			Standard	High-residue	<0.001
		High-residue	7.36	7.27	6.91	7.96	0.44	0.35			Basic	High-residue	<0.001
4	iron sulfate	Standard	6.87	7.07	4.96	9.44	1.47	1.34	9.90	<0.001	Standard	Basic	>0.01
		Basic	5.50	5.23	4.92	6.56	0.85	0.62			Standard	High-residue	>0.01
		High-residue	5.17	5.15	4.98	5.49	0.29	0.18			Basic	High-residue	>0.05
5	iron fumarate	Standard	1.92	1.85	1.63	2.21	0.36	0.22	97.44	<0.001	Standard	Basic	<0.001
		Basic	3.65	3.67	2.99	3.98	0.38	0.31			Standard	High-residue	<0.001
		High-residue	3.50	3.34	3.10	3.90	0.58	0.33			Basic	High-residue	<0.05
6	iron bisglycinate—Ferrochel^®^ TRAACS^®^ (iron amino acid chelate)	Standard	9.77	9.55	8.31	11.24	1.82	1.07	64.08	<0.001	Standard	Basic	<0.001
		Basic	6.25	6.41	5.74	6.72	0.81	0.42			Standard	High-residue	<0.001
		High-residue	5.92	5.93	4.65	7.18	0.71	0.77			Basic	High-residue	>0.05
7	iron gluconate	Standard	8.18	8.37	6.95	9.41	2.03	1.05	9.62	<0.001	Standard	Basic	<0.01
		Basic	10.00	9.82	7.91	12.09	0.68	1.15			Standard	High-residue	>0.05
		High-residue	8.59	8.71	7.99	8.96	0.53	0.35			Basic	High-residue	<0.01
8	iron sulfate	Standard	21.16	22.18	17.99	24.33	4.75	2.51	10.85	<0.001	Standard	Basic	<0.01
		Basic	18.59	18.78	17.98	19.09	0.57	0.41			Standard	High-residue	<0.001
		High-residue	18.17	18.16	17.82	18.53	0.13	0.22			Basic	High-residue	<0.05

**Table 4:** The bioaccessibility (%) of iron considering chemical form under the influence of various types of diets.

Chemical Form	Diet	*n*	M	Me	Min	Max	IQR	SD	One-Way ANOVA		Tukey’s Post Hoc Test Results		
									F	*p*	Group 1	Group 2	*p*
Iron fumarate	Standard	27	3.06	2.77	2.77	6.41	1.96	1.28	22.06	<0.001	Standard	Basic	<0.001
	Basic	27	9.19	7.39	7.39	17.58	11.63	5.46			Standard	High-residue	<0.05
	High-residue	27	5.83	6.68	6.68	7.96	3.45	1.76			Basic	High-residue	<0.01
Iron lactate	Standard	9	4.56	4.62	3.92	5.13	0.75	0.42	9.30	<0.001	Standard	Basic	<0.001
	Basic	9	6.02	6.34	4.20	7.84	1.40	1.10			Standard	High-residue	>0.05
	High-residue	9	5.16	5.28	4.55	5.76	0.61	0.43			Basic	High-residue	<0.05
Iron sulfate	Standard	18	14.02	13.72	4.96	24.33	15.11	7.61	0.58	>0.05	Standard	Basic	>0.05
	Basic	18	12.04	12.27	4.92	19.09	13.55	6.75			Standard	High-residue	>0.05
	High-residue	18	11.67	11.66	4.98	18.53	13.01	6.69			Basic	High-residue	>0.05
Iron bisglycinate (iron amino acid chelate—Ferrochel^®^ TRAACS^®^)	Standard	9	9.77	9.55	8.31	11.24	1.82	1.07	64.08	<0.001	Standard	Basic	<0.001
	Basic	9	6.25	6.41	5.74	6.72	0.81	0.42			Standard	High-residue	<0.001
	High-residue	9	5.92	5.93	4.65	7.18	0.71	0.77			Basic	High-residue	>0.05
Iron gluconate	Standard	9	8.18	8.37	6.95	9.41	2.03	1.05	9.62	<0.001	Standard	Basic	<0.01
	Basic	9	10.0	9.82	7.91	12.09	0.58	1.15			Standard	High-residue	>0.05
	High-residue	9	8.59	8.71	7.99	8.96	0.53	0.35			Basic	High-residue	<0.01

**Table 5:** The bioaccessibility (%) of iron, considering only the chemical form.

Chemical Form	*n*	M	Me	Min	Max	IQR	SD	Kruskal–Wallis ANOVA	
								H	*p*
Iron fumarate	81	6.03	4.38	1.51	17.58	4.09	4.20	71.63	<0.001
Iron lactate	27	5.25	5.03	3.92	7.84	1.17	0.92		
Iron sulfate	54	12.58	13.63	4.92	24.33	13.11	6.97		
Iron bisglycinate (iron amino acid chelate—Ferrochel^®^ TRAACS^®^)	27	7.31	6.58	4.65	11.24	3.15	1.93		
Iron gluconate	27	8.92	8.94	6.95	12.09	1.50	1.19		
Diet without preparation	27	6.36	6.69	3.31	9.27	2.96	1.68		

H—test statistic value for the ANOVA test.

**Table 6:** The average bioaccessibility (%) of iron, considering the pharmaceutical form.

Pharmaceutical Form	*n*	M	Me	Min	Max	IQR	SD	Kruskal–Wallis ANOVA	
								H	*p*
Diet without preparation	27	6.36	6.69	3.31	9.27	2.96	1.68	53.99	<0.001
Coated tablets	135	7.02	6.51	1.51	17.58	2.98	3.17		
Tablets	27	3.02	3.29	1.63	3.98	1.68	0.85		
Extended-release tablets	27	19.31	18.46	17.82	24.33	0.94	1.96		
Capsules	27	7.31	6.58	4.65	11.24	3.15	1.93		

## Data Availability

The original contributions presented in this study are included in the article/Supplementary Materials. Further inquiries can be directed to the corresponding author.

## Supplementary Materials

The following supporting information can be downloaded at https://www.mdpi.com/article/10.3390/nu18081219/s1. Table S1: Selected nutritional parameters of diets used in the study; Table S2: Composition of diets used in the study; Table S3: Detailed composition of dietary supplements and medicinal products used in the experiment; Table S4: Operating parameters in the ICP-OES method.
