# Supplementary material for: Influence of Diet on Bioaccessibility of Iron from Dietary Supplements and Medicinal Products—Results of In Vitro Digestion Model and Analytical Determinations
Source: Nutrients. 2026 Apr 13;18(8):1219. doi: 10.3390/nu18081219 (PMC13118478; doi:10.3390/nu18081219)
Supplement: Supplementary file 1 [file nutrients-18-01219-s001.zip › nutrients-4225881-supplementary.pdf]

SUPPLEMENTARY FILE

Influence of Diet on Bioaccessibility of Iron from Dietary Supplements and Medicinal Products—Results of In Vitro Digestion Model and Analytical Determinations

Table S1. Selected nutritional parameters of diets used in the study.

Parameter	Diet		
	Basic	Standard	High-residue
Proteins (g)	116.8 (17.3% of E*)	153.9 (22.2% of E)	143.5 (18.5% of E)
Fats (g)	109.2 (36.4% of E)	129 (41.9% of E)	117.1 (34% of E)
Carbohydrates (g)	338 (46.3% of E)	272.2 (35.8% of E)	416 (47.5% of E)
Fiber (g)	28.6	24.6	50.2
Vitamin A (μg)	2960	500	4600
Vitamin C (μg)	80.6	54.8	304.7
Vitamin E (mg)	17.2	15.9	24.7
Calcium (mg)	568.7	1203	1465
Sodium (mg)	2370	4865	3290
Potassium (mg)	4877	5615	6837
Magnesium (mg)	361	562.9	693

Iron (mg)	14.3	17.2	21.7
Energy (kcal)	2699	2770	3099
Total weight (g)	2970	2750	3285

* Percentage of energy

Table S2. Composition of diets used in the study.

Type of the meal	Food product/meal	Amount (g/mL)
**Basic diet**		
Breakfast	cereal coffee with milk	250
	white bread	80
	cottage cheese	110
	Onion	10
	cucumber	30
	butter	10 g
Second breakfast	mixed bread (wheat-rye flour)	60
	chicken pate	130
	apple	150
	black tea infusion	250

Lunch	vegetable soup (including carrots, celery, parsley, cauliflower)	400 (vegetables – 100 g)
	poultry chop	150
	potatoes	300
	red cabbage salad	150
	grated strawberry compote	250 (strawberries -30 g)
Dinner	meatballs in sauce	120
	pasta	120
	salad (carrot, apple, mayonnaise)	100
	yeast cake with crumble	50
	black tea infusion with milk	250
**Standard diet**		
Breakfast	ham sausages	150
	mixed bread (wheat-rye flour)	90

	cocoa with milk	250
	mustard	20
Second breakfast	gouda cheese	60

	crispbread	30
	coffee infusion with milk	150
Lunch	tomato soup with pasta	400
	Potatoes	300
	grilled cod	200
	salad with sauerkraut	150
	compote	250
	coffee infusion with milk	150
	milk chocolate with nuts	30
Dinner	mixed bread (wheat-rye flour)	80
	sausages	110
	pickled cucumber	80
	black tea infusion	250
**High-residue diet**		
Breakfast	oatmeal in milk	oat flakes – 50 g
		milk – 350 g
	wholemeal bread	80

	cottage cheese	80
	jam	20
Second breakfast	mixed bread (wheat-rye flour)	80
	rennet cheese	40
	ham	440
	tomatoes	150
	banana	100
	black tea infusion	250
Lunch	beetroot soup	400 (beetroot – 30 g; vegetables: carrots, parsley, celery – 25 g)
	pork chop	150
	potatoes	300
	boiled vegetables (carrots with peas 1+1 with breadcrumbs and butter)	300
	apple	150
	strawberry compote	200
Dinner	mixed bread (wheat-rye flour)	80
	chicken ham	80

	red pepper	150
	butter	10 g
	coffee infusion with milk	150

Table S3. Detailed composition of dietary supplements and medicinal products used in the experiment.

Product No	Composition
1	magnesium hydroxide, ginseng root extract (Panax ginseng C. A. Meyer) standardized to 8% ginsenosides, L-ascorbic acid, bulking agents (microcrystalline cellulose, calcium phosphates, sodium carboxymethylcellulose, cross-linked), lecithin (from soy), ferrous fumarate, maltodextrin, corn starch, glazing agents (magnesium salts of fatty acids, hydroxypropyl methylcellulose, shellac, polydextrose, talc, polyethylene glycol, carnauba wax), DL-α-tocopheryl acetate, nicotinamide, zinc oxide, calcium D-pantothenate, colours (titanium dioxide, iron oxides and hydroxides), humectant (glycerol), copper (II) sulfate, pyridoxine hydrochloride, riboflavin, thiamine mononitrate, retinyl acetate, pteroylmonoglutamic acid, potassium iodide, sodium selenate (IV), D-biotin, cholecalciferol, cyanocobalamin
2	bulking agent: cellulose, L-ascorbic acid, glazing agent: hydroxypropylmethylcellulose, sodium selenate, zinc sulphate, iron (II) lactate, nicotinamide, glazing agent: talc, anti-caking agent: silicon dioxide, calcium D-pantothenate, glazing agents: magnesium salts of fatty acids, DL-alpha-tocopheryl acetate, colour: titanium dioxide, cholecalciferol, marigold flower extract, pyridoxine hydrochloride, glazing agent: hydroxypropylcellulose, riboflavin, thiamine mononitrate, pteroylmonoglutamic acid, potassium iodide, D-biotin, colour: quinoline yellow, cyanocobalamin, colour: sunset yellow FCF, glazing agents: beeswax and carnauba wax
3	Calcium carbonate (calcium); bulking agent: microcrystalline cellulose; magnesium oxide (magnesium); L-ascorbic acid (vitamin C); binding agent: polyvinylpyrrolidone; bulking agent: cross-linked sodium carboxymethylcellulose; nicotinamide (niacin); iron (II) fumarate (iron); glazing agent: hydroxypropylmethylcellulose; DL-alpha-tocopheryl

	acetate (vitamin E); color: titanium dioxide; glazing agent: polydextrose; zinc oxide (zinc); anti-caking agent: magnesium stearate; calcium D-pantothenate (pantothenic acid); glazing agents: talc, maltodextrin, medium-chain triglycerides; riboflavin (vitamin B2); Pyridoxine hydrochloride (vitamin B6); thiamine mononitrate (vitamin B1); retinol acetate (vitamin A); manganese sulfate H2O (manganese); pteroylmonoglutamic acid (folic acid); color: carmine acid; potassium iodide (iodine); D-biotin; phytoquinone (vitamin K); chromium picolinate (chromium); sodium molybdate (molybdenum); sodium selenite (selenium); cholecalciferol (vitamin D); cyanocobalamin (vitamin B12).
4	bulking agent: microcrystalline cellulose; L-ascorbic acid (vitamin C); iron (II) sulfate; zinc sulfate; potassium iodide; nicotinamide (niacin); calcium D-pantothenate (pantothenic acid); DL-alpha tocopheryl acetate (vitamin E); anti-caking agent: magnesium salts of fatty acids; lutein; pyridoxine hydrochloride (vitamin B6), riboflavin 5′-phosphate sodium (vitamin B2); thiamine mononitrate (vitamin B1); rutin; pteroylmonoglutamic acid (folic acid); D-biotin; cyanocobalamin (vitamin B12); coating (thickener: hydroxypropylmethylcellulose, color: calcium carbonate, binder: talc, bulking agent: microcrystalline cellulose, colors: E171 and E172)
5	Bulking agent: microcrystalline cellulose; caffeine; magnesium oxide; L-ascorbic acid; bulking agent: calcium phosphates (dicalcium phosphate); citrus bioflavonoids; guarana seed extract (Paullinia cupana) standardized to 22% caffeine; nicotinamide; ginseng root extract (Panax ginseng); glazing agents: fatty acids, hydroxypropyl methylcellulose; potassium iodide; calcium D-pantothenate; color: calcium carbonate; methylcobalamin; glazing agents: magnesium salts of fatty acids, silicon dioxide; pyridoxine hydrochloride; riboflavin; thiamine mononitrate; glazing agent: polyethylene glycol; color: iron oxides and hydroxides; pteroylmonoglutamic acid; anti-caking agent: talc
6	maltodextrin, microcrystalline cellulose – bulking agent, iron bisglycinate (iron amino acid chelate Ferrochel® TRAACS®), L-ascorbic acid (vit. C), magnesium stearate – anti-caking agent, pteroylmonoglutamic acid (folate), pyridoxine hydrochloride (vit. B6), cyanocobalamin (vit. B12), capsule (gelatin – shell component, colour: E 171)
7	Active substance: iron (II) gluconate 200 mg, equivalent to 23.2 mg of iron ions. Excipients:

	ascorbic acid, talc, potato starch, stearic acid, propylene glycol, and AquaPolish P red coating composed of: hypromellose, hydroxypropylcellulose, macrogol, medium-chain triglycerides, talc, titanium dioxide (E 171), Allura Red AC lake (E 129), black iron oxide (E 172), red iron oxide (E 172)
8	Active substance: iron (II) ions in the form of dried iron (II) sulfate. Excipients: lactose monohydrate, ammonio methacrylate copolymer, talc, magnesium stearate, sucrose, gum arabic, gelatin, cochineal red (E 124)

Table S4. Operating parameters in the ICP-OES method.

Analytical line, reading time	Fe 259.940 nm, 6 s
Signal reading type	Radial
Signal integration	3 pix
Plasma generator power	1300 W
Coolant gas flow rate	14 $L{\cdot}min^{-1}$
Auxiliary gas flow rate	0.5 $L{\cdot}min^{-1}$
Carrier gas flow rate	0.6 $L{\cdot}min^{-1}$
Sample flow rate	1.0 $L{\cdot}min^{-1}$
